# A Case Study of the *De Novo* Evolution of a Complex Odometric Behavior in Digital Organisms

**DOI:** 10.1371/journal.pone.0060466

**Published:** 2013-04-08

**Authors:** Laura M. Grabowski, David M. Bryson, Fred C. Dyer, Robert T. Pennock, Charles Ofria

**Affiliations:** 1 Department of Computer Science, University of Texas-Pan American, Edinburg, Texas, United States of America; 2 BEACON Center for the Study of Evolution in Action, Michigan State University, East Lansing, Michigan, United States of America; University of Sussex, United Kingdom

## Abstract

Investigating the evolution of animal behavior is difficult. The fossil record leaves few clues that would allow us to recapitulate the path that evolution took to build a complex behavior, and the large population sizes and long time scales required prevent us from re-evolving such behaviors in a laboratory setting. We present results of a study in which digital organisms–self-replicating computer programs that are subject to mutations and selection–evolved in different environments that required information about past experience for fitness-enhancing behavioral decisions. One population evolved a mechanism for step-counting, a surprisingly complex odometric behavior that was only indirectly related to enhancing fitness. We examine in detail the operation of the evolved mechanism and the evolutionary transitions that produced this striking example of a complex behavior.

## Introduction

Like structural and physiological traits, behavioral traits are an essential aspect of the biology of animals, and elicit similar evolutionary questions. However, investigating the evolution of animal behavior is slowed by the fact that behavior leaves little evidence in the fossil record, and can generally be observed only in living specimens under restricted conditions of the sort they would experience in nature. Despite major advances during the last century in studying and understanding animal behavior, determining how behavioral traits evolved is, because of such reasons, limited to examining the most recent periods in the evolution of behavior. A promising new approach avoids these limitations using *digital evolution* models, which instantiate evolutionary causal processes in a virtual environment so that hypotheses about the evolution of behavior functions can be tested experimentally. Studying the evolution of complex behavior in a virtual world offers the opportunity to observe behavioral evolution in action, from simple capabilities to complex behavioral repertoires. We can also examine the details of underlying genetic structure to discern its relationship to behavior, an option that is available only rarely and to a highly limited degree when studying behavior in living specimens.

In this paper, we perform a case study that explores the evolutionary history of an example complex behavior. The details of this history provide insight into how evolution produces complex behavior where no complexity existed before. Our results also support theoretical views of how complexity evolves in nature. The specific behavior that evolved in our experiments was the ability to count steps in order to track distance traveled. This behavior arose in open-ended evolution in experiments conducted in the Avida system [1], in an environment where neither distance tracking nor counting was directly selected for, but may have provided a behavioral solution to certain environmental challenges. Results of related experiments have been reported in [2], [3]. The current discussion focuses on the evolved step-counting mechanism and the evolutionary transitions that led to that mechanism. The evolved odometric behavior appeared in only one population; other populations evolved different survival strategies. Despite its rarity, the step-counting capacity is a proof of principle and is sophisticated enough to warrant examination as a case study for the evolution of complex behaviors.

Many researchers are keenly interested in the evolution of biological complexity, and have approached this issue from a number of perspectives. Adami *et al.* [4] applied information theory to measure evolving genomic complexity in digital organisms. They showed that genomic complexity increases under the action of natural selection, as “informative” mutations (*i.e.*, mutations that increase an organism's ability to survive) are preserved. Goldstein [5] explored the evolution of multicellularity from the perspective of physics, hypothesizing a connection between the transition to multicellularity and competing aspects of fluid dynamics. Lenski *et al.* [6] used the Avida system to demonstrate the evolution of complex features that originated from random mutations and natural selection, building complex functions from simple functions that evolved earlier. This work by Lenski and colleagues is particularly relevant to our paper, and provides a definition of complexity for Avida organisms. In general, a function in Avida is complex if it requires the coordinated execution of several individual instructions. More than one mutation from the original ancestor organism are needed for any such sequence of instructions, and the order of execution of the instructions must be correct (in terms of accomplishing the particular function) and consistent. In Lenski *et al.* [6], for example, the most complex logic function, logical equals (EQU) required a program at least 19 instructions long. In this paper, we examine another sequence of instructions that produced behavior analogous to step-counting.

Within the context of biology, animal behavior provides many examples of complexity. One such example is navigation. Even seemingly simple animals accomplish sophisticated and complex navigation tasks. A number of models have been proposed for a variety of animal navigation behaviors, and several approaches used *in silico* evolution. Among these models, Dale and Collett [7], Vickerstaff and Di Paolo [8], and Haferlach *et al.* [9] are perhaps the most relevant to the current discussion. Dale and Collett [7] used an evolutionary algorithm to evolve motor controllers for an “animat,” or artificial animal, and compared the artificially evolved navigation strategies to those of flying insects, specifically wasps and bees. Their results suggested that insects' navigation strategies are mostly adaptations to the demands of reaching a spatial position using visual information and compass directions. Vickerstaff and Di Paolo [8] focused on modeling the homing behavior of the Saharan desert ant, *Cataglyphis fortis*, by using a genetic algorithm to evolve a neural model of path integration. Their results produced the same sort of systematic errors observed in the desert ants and the systematic searching behavior the ants perform once they reach the estimated nest location. Haferlach *et al.* [9] also used a genetic algorithm to evolve a neural model of path integration, using biologically plausible direction cells, with input encoded in a manner analogous to polarization-sensitive insect interneurons. Studies such as these provide interesting and valuable insights, but it should be noted that they differ from the current study in several key aspects. First, the experiments for the current case study were not designed to test a hypothesis related to navigation. Instead, the experiments focused on the evolution of memory use, with navigation providing only the context for the experiments. Second, the behavior that we are examining in this paper, *i.e.*, step-counting, was not directly selected for during evolution, and in fact its appearance was a complete surprise. Third, the experiments that gave rise to the case study organism discussed in this paper were performed using a different underlying representation (*i.e.*, a genetic program) and a different type of methodology, namely digital evolution. Studies similar to [7]–[9] that explicitly test hypotheses about animal behavior can be performed using the Avida system. A recent example of such a study is Goldsby *et al.* [10], in which the authors tested the hypothesis that the evolution of temporal polytheism in eusocial insects relates to pressures resulting from the relative risk associated with various tasks and aging. Their results demonstrate the Avida experiments can proceed biologically relevant insights into behavior despite the differences between the Avida organisms and living ones.

Odometry, *i.e.*, measuring distance traveled, is an important aspect of certain navigation behavior. Here, we use the term odometry to refer to measuring distance only, as opposed to integrated measures of distance such as path integration. Navigation behavior in ants is well studied, and many sensory and behavioral components of their navigation behaviors have been described in detail, for example vector navigation [11], landmark use [12], and compasses for navigation [13]. Odometry mechanisms in ants are less well explored (for a review, see [14]), but evidence exists [15] for “stride integration,” combining the length and number of steps to measure distance traveled. This idea of measuring distance traveled using self-movement provides the context for our case study.

The remainder of the paper is organized as follows. We present an overview of the Avida system and some details of the experiments for the case study. We then present the results of the experiments, first explaining in detail the evolved behavior itself and then how the digital organism's genome produced that behavior. We then provide an in-depth analysis of how the structure and function of the step-counting section of the organism's genome emerged over evolutionary time.

## Materials and Methods

Digital evolution [4] places a population of self-replicating computer programs in a computational environment. The population evolves as these “digital organisms” compete for resources, replicate, and mutate. Digital evolution approaches furnish tools for investigating evolutionary processes in biology, and for harnessing the power of evolution to develop solutions to computing and engineering problems. Avida [1] is a widely used digital evolution software platform and has repeatedly provided insights into evolutionary processes, including effects of mutation rates [16], [17], sexual reproduction and genetic architecture [18], inclusive fitness theory [19] and, of course, the evolution of complexity [4], [6].

The Avida world contains a population of digital organisms. The size of the world remains fixed throughout the duration of an experiment, limiting the number of individuals in the population. Each individual Avida organism, or “Avidian,” is composed of its genome (a circular list of program instructions that are similar to assembly language) and a virtual central processing unit (CPU) that executes the instructions in the organism's genome. The default CPU has three general-purpose registers (AX, BX, and CX), two stacks, and several heads (IP, the instruction pointer; FLOW, a target for jumps; READ and WRITE, targets for copying). Execution of the organism's instructions acts on the elements of its virtual CPU, and instruction execution incurs a cost measured in virtual CPU cycles. An Avidian must execute instructions to perform any function, including replication, movement, and sensing. The basic set of Avida instructions is Turing-complete [20] and the existing capabilities of the system may be extended by adding new instructions.

An Avidian replicates by copying the instructions in its genome into a block of memory that becomes its offspring. During the copying process, errors may occur that result in differences between the genomes of parent and offspring (*i.e.*, mutations). A new offspring is produced when the parent organism has finished copying its genome and divides. The new offspring is placed in a random cell on the grid, replacing any organism that was occupying that cell. Thus, an organism that replicates more quickly than another will tend to have more descendants in future populations. Avidians may replicate sooner if they perform user-defined tasks that speed up their execution rate. When an Avidian performs a task, it will receive a bonus that will increase its future metabolic rate, allowing it to execute more instructions in a unit of time than an organism with a lower metabolic rate [1].

Avida genomes may grow or shrink due to insertion or deletion mutations and selective pressures. This approach allows organisms to extend their genomes to allow for more complex instruction sequences that may be required for more complex tasks. An organism's initial execution speed is proportional to the length of its genome, so organisms that increase in length are not penalized solely for having a longer genome.

Our experimental environments were inspired by maze-learning experiments with honey bees [21], in which the bees learned to follow different visual cues through a maze to a food goal. Similar to the bee maze experiments, in our experiments each digital organism had its own isolated environment (called a “state grid”) containing a path that it could follow to gather food and increase the rate of its metabolism (the rate at which it executed genomic instructions). An organism needed to gather sensory information from the environment and react appropriately to cues. The experiments were designed to explore the evolution of memory use, and effective strategies in these environments required the evolution of different ways of storing and reusing experience. The evolved organisms were able to take sensory input from their environment and make behavioral decisions based on that input. Organisms were able to react consistently to sensory cues from the environment, and were able to remember past experience in making new behavioral choices [2], [3].

We added three types of new instructions for movement, sensing, and comparison to the basic Avida instruction set for our experiments. (1) *Movement.* The sg-move instruction enables an organism to move one cell in the direction of its current orientation in the state grid. Orientation changes are accomplished by sg-rotate-right and sg-rotate-left, instructions that change the organism's orientation by 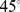 in the specified direction. (2) *Sensing.* The sg-sense instruction places a value into one of the organism's virtual registers, based on the contents of its current grid cell. Specific values are described below. (3) *Comparisons.* We supplemented the basic Avida instruction set with new comparisons, if-grt-X (*if greater than X*) and if-equ-X (*if equal to X*) that permit an organism to compare the contents of its BX register with a set value. The value used for the comparison is determined by a NO-OP (nop) label immediately following the comparison instruction and thus could be directly evolved as a constant. The new instructions provided the Avidians a simple mechanism for comparisons with constants, while allowing for simpler evolved genomes.

Each environment in the experiments that evolved the case study organism contained some combination of the following cues:

1. **Nutrient:** A cue that indicates the current cell is on the path, and provides energy that adds to the organism's metabolic bonus (the “food” on the path). This cue has a sense input value of 0 with the sg-sense instruction.
2. **Directional cue:** A cue that indicates to turn either right or left (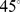 in the specified direction) in order to remain on the path. Cells with directional cues also contain nutrient. The sg-sense instruction returns 2 for right and 4 for left turns.
3. **Empty:** A cue that indicates cells that are off the path. Movement into an empty cell depletes energy that an organism gains by moving into a cell on the path. The sg-sense instruction returns -1 for empty cells.

Other environments were used for additional experiments, but are not relevant to the current case study [2], [3]. The environments for the case study were the simplest of all the experimental environments, containing only right turns or left turns. We used these environments for two reasons. First, in such an environment, an Avidian needed to sense and react to the minimum number of cues in any one environment (nutrient, one type of turn cue, and empty), reducing the overall complexity of the task. Second, these environments are analogous to the “continuous turn” environments of the Zhang *et al.* experiments [21] that provided the inspiration for our experimental environments.

The metabolic rate bonus an organism received was determined by how well it followed the path. An organism that traversed the entire path without stepping off received the maximum bonus. The bonus calculation is based on the number of unique path cells an organism encountered less the total number of movements into cells that are off the path (a negative value is not permitted). Organisms were not penalized for taking extra steps on the path, but neither did they receive additional credit. The calculated bonus doubled for each step on the path that was not counteracted by a movement into an empty cell off the path. Refer to [2], [3] for additional experiment details and background information.

During evolution, organisms were presented with one of four different paths, selected randomly. Two paths contained only right turns and two contained only left turns. Each individual experienced only one path in its lifetime, and different paths could be experienced by different organisms in the same generation.

We ran 50 experimental populations with a maximum of 3600 individuals for 250,000 updates (the native time unit in Avida, approximately 11,200 generations in the case study population). Each population was seeded with an organism that had only the ability to replicate. The ancestor organism's genome is 100 instructions long and includes replication-related instructions and a large number of nop instructions. Other instructions and functions may appear only through mutations. Our experiments used a 0.085 genomic mutation rate for a length-100 organism (0.0075 copy mutation probability per site, insertion and deletion probabilities of 0.05 per divide) [1].

## Results and Discussion

In this section, we present the results of the experiments for the case study organism. The discussion is organized in three parts: (1) **Evolved Behavior** describes the observed behavior of the case study organism in test environments, (2) **Evolved Odometry** provides a detailed instruction-by-instruction analysis of how the evolved step-counting mechanism operates, and (3) **Evolving the Step Counter** examines the evolutionary history of case study organism from the initial ancestor.

### Evolved Behavior

**Figure 1:**
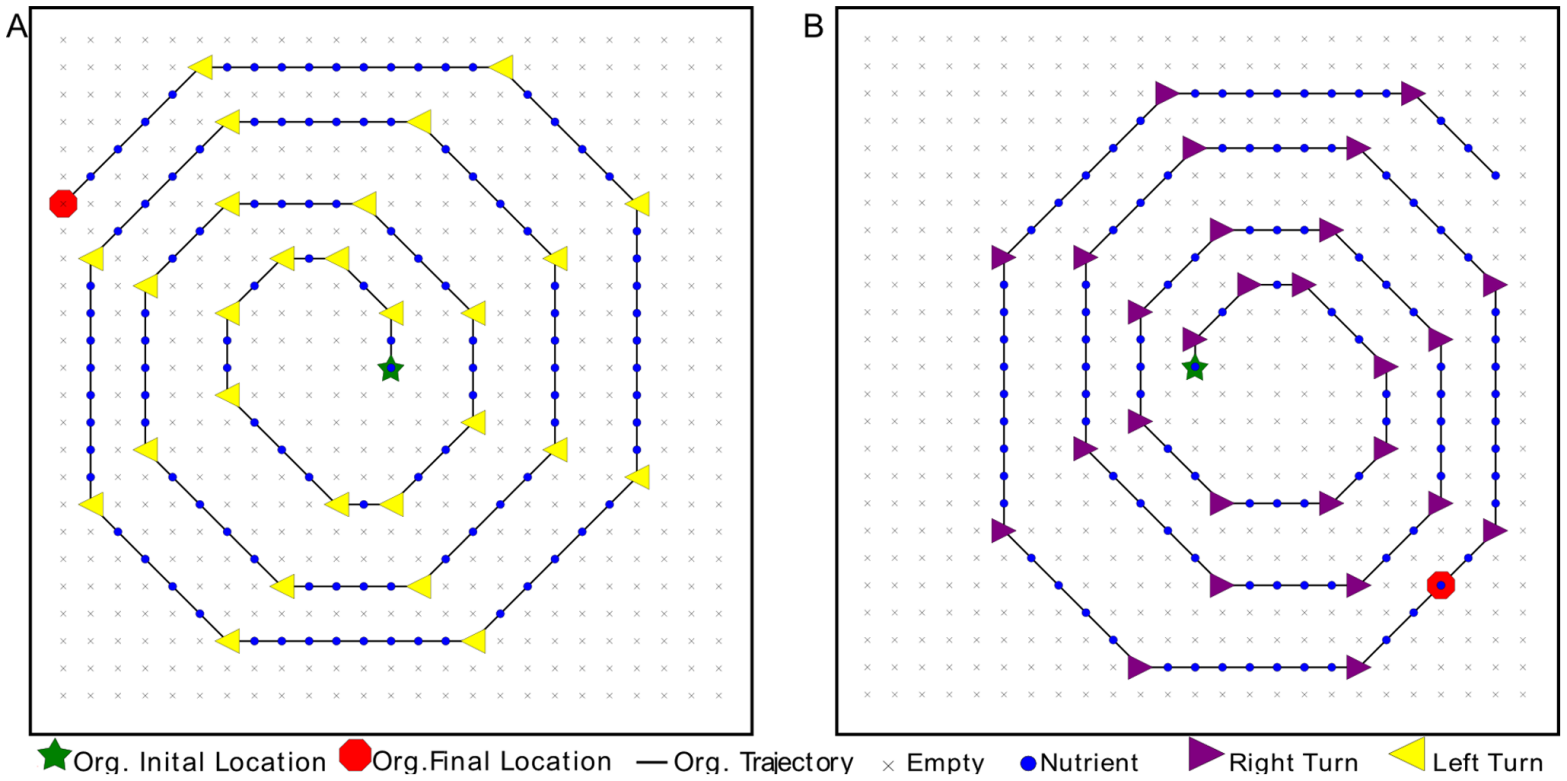
Trajectories of evolved case-study organism on paths experienced during evolution. The organism's trajectory is shown with the black line, beginning at the green star and ending at the red octagon (note: in some cases the organism doubles back along its own path). On the left-turn path (**A**), the organism traverses the path, steps off the end and stops to finish replication. On the right-turn path (**B**), the organism has retraced its steps from the end of the path to its final location (red octagon).

When functioning in an environment where all cues signal left-turns, the evolved case study organism traversed the length of the path, stepping exactly one cell off the end of the path and stopping (Figure 1A). This strategy gave a high metabolic rate bonus, since the organism took only one step off the path. We thought that this type of strategy was the best that an organism could do, since there is no way for it to detect the next cell except by stepping into it and sensing. Indeed, this was the observed strategy that evolved in other populations in several different experimental setups [2], [3]. However, in right-turn environments, the case study organism somehow improved on its left-turn paths performance, navigating the path without moving into any empty cells (Figure 1B), earning it all possible metabolic bonuses, with no penalties at all. This surprising result spurred us to investigate how this outcome was possible.

Since the ancestral environments (*i.e.*, the environments experienced during evolution) were all similar, we tested how the behavior generalizes in similar but novel environments. These environments have the same fundamental features as the ancestral environments, but turns occurred at different positions, path lengths differed, and the specific trails were never experienced during evolution. We placed the organism in several different novel environments: (1) an ancestral left-turn path that was modified by extending the end of the path; (2) a completely unfamiliar left-turn environment; (3) an ancestral right-turn path with an extended end; and (4) an unfamiliar right-turn environment.

**Figure 2:**
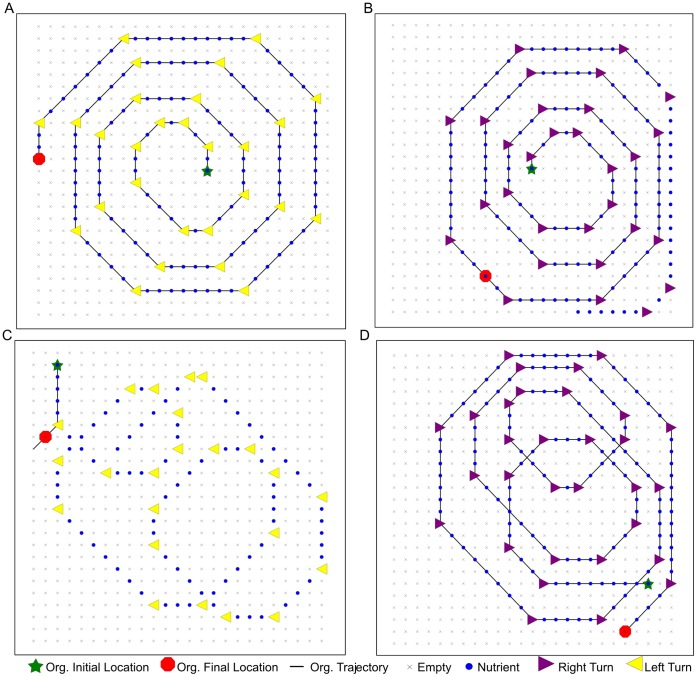
Example trajectories of the step-counting organism traversing unfamiliar paths. (**A**) Modified ancestral left-turn path, with extended path end. The organism successfully travels the entire path, stepping exactly one cell off the end and then stopping. (**B**) Modified ancestral right-turn path, with extended path end. The organism turns and retraces its steps before reaching the path end. (**C**) Unfamiliar left-turn path. The organism turns incorrectly at the first turn. The organism neither traverses the path nor replicates in this environment. (**D**) Unfamiliar long right-turn path. The organism traverses the entire path but fails to self-replicate.

Figure 2 shows the organism's performance in each of the above conditions. The results reveal the key feature of this organism's algorithm (first described in [3]): the organism counts the number of steps it has traveled on a right-turn path and turns around at a specific distance. The organism is able to follow the extended right-turn path of Figure 2B, but it uses its step-counting mechanism and turns around before it completes this modified ancestral path. The organism also manages to navigate the extended left-turn path (Figure 2A); its behavior is qualitatively identical to its behavior when traversing the original ancestral path. The unfamiliar right-turn path (Figure 2D) is longer than any ancestral path and the extended right-turn path in Figure 2B. The organism appears to traverse the entire path, stopping after one step off the end of the path; in fact, the organism fails to replicate in this environment. This result reveals that the step-counting mechanism does not function reliably in all right-turn environments, but is crucial to replication in those environments. The organism's strategy also fails in the unfamiliar left-turn environment (Figure 2C); the organism turns the wrong direction at the first turning, takes a few steps in empty cells, and stops moving, once again without completing replication. This behavioral evidence suggests that the evolved strategy is brittle in unfamiliar environments. The explanation for the differences in behavior is found through a closer examination of the step-counting mechanism.

### Evolved Odometry

To understand the odometry mechanism and trace its evolutionary origin, we need to look at the level of the genome. For this analysis, we traced the organism's execution in both right- and left-turn environments. These traces contain detailed information about the state of the organism's virtual CPU elements, the sensory input from the environment, and the movement history of the organism. These details allow us to observe the moment-by-moment execution of the organism's program.

The evolved organism is 185 instructions long, with an executed size of 156 instructions. As discussed in [3], most (but not all) of the critical code of this organism is organized in two modules: one loop that contains the step-counting routine and handles much of the movement for right-turn situations, and another that handles moving with left turns and has a nested copy loop for replication. The 16-instruction step-counting module is located at genome sites 117–132. Table 1 shows this part of the organism's genome, detailing each instruction and its effect when executed. Table 2 provides an example excerpt of the execution flow of the evolved organism's genome.

**Table 1:** Detail of evolved counting organism's genome.

Site	Instruction	Instruction Functionality
117	h-search	Marks the start of counting module.
118	sg-rotate-r	Turn right 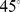 .
119	if-grt-0	CX register contents 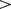 0?
120	nop-C	This comparison is TRUE when on a right-turn path.
121	h-copy	Copy when (executes only when on a right-turn path).
122	h-copy	Copy (always executes).
123	sg-sense	Put current sense input into CX register.
124	nop-C	
125	jmp-head	Move the IP the number of instructions designated by the value in CX register.
		If sense input was nutrient, CX = 0; IP does not change.
		If sense input was right, CX = 2; IP skips 126–127 and moves to site 128.
		If sense input was left, CX = 4; IP skips126–129 and moves to site 130.
126	sg-rotate-l	Executes only when sense input is nutrient (*i.e.*, CX = 0).
		When executed, undoes right turn at top of loop.
127	if-equ-X	BX register contents = 1?
		This comparison is TRUE on a right-turn path.
128	get-head	Put the current value of IP into CX (*i.e.*, CX = 128).
		Executes only when on a right-turn path.
129	sg-move	Take a step. Does not execute on a left-turn path.
130	inc	Increment value in BX (*i.e.*, BX = BX+1).
131	if-n-equ	If contents of BX are not equal to contents of CX, execute the next instruction.
		Instruction tests for loop exit conditions.
132	mov-head	Exit on right-turn path after taking 127 steps, then incrementing BX to 128.
		Exit on left-turn path after turning 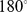 without taking any steps.

Detail of the instructions in the counting module of the evolved counting organism's genome. **Site** refers to the position in the genome of the instruction, **Instruction** is the specific Avida instruction, and **Instruction Functionality** describes how the instruction operates.

**Table 2:** Example execution segment.

Exec	Site	Instruction	Facing	Flow	BX	CX	Remarks
95	117	h-search	N	**117**	**1**	**0**	Move flow-control head to current position
96	118	sg-rotate-r	**NE**	**117**	**1**	**0**	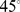 Turn right
97	119	if-grt-0	NE	117	1	0	CX register contents 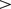 0? No! Skip next instruction.
98	**122**	**h-copy**	**NE**	**117**	**1**	**0**	Copy instruction (regardless of path)
99	123	sg-sense	NE	117	1	**0**	Put 0 in CX (We are currently on a nutrient)
100	125	jmp-head	NE	117	1	0	Jump ahead CX instructions (in this case, don't move)
101	**126**	**sg-rotate-l**	**N**	**117**	**1**	**0**	Turn left; compensate for the previous right turn to go straight
102	127	if-equ-X	N	117	1	0	By default X = 1; Since BX is 1, execute next instruction
103	128	get-head	N	117	1	**128**	Put the current value of IP into CX (*i.e.*, CX = 128)
104	129	sg-move	N	117	1	128	Take a step onto the right turn cue
105	130	inc	N	117	**2**	**128**	Increment value in BX (*i.e.*, BX = BX+1).
106	131	if-n-equ	N	117	2	128	BX ! = CX (yet), so execute the next instruction.
107	132	mov-head	N	117	2	128	Restart loop by jumping to flow head (117)
108	**117**	**h-search**	**N**	**117**	**2**	**128**	Would move flow head, but already there
109	118	sg-rotate-r	**NE**	**117**	**2**	**128**	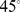 Turn right
110	119	if-grt-0	NE	117	2	128	CX register contents 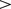 0?
111	121	h-copy	NE	117	2	128	Copy instruction (since we're on a right-turn path)
112	122	h-copy	NE	117	2	128	Copy instruction (regardless of path)
113	123	sg-sense	NE	117	2	**2**	Put 2 in CX (We are currently on a right-turn cue)
114	125	jmp-head	NE	117	2	2	Jump ahead CX instructions (*i.e.*, move to 128 instead of 126)
115	**128**	**get-head**	**NE**	**117**	**2**	**128**	Put the current value of IP into CX (*i.e.*, CX = 128)
116	129	sg-move	NE	117	2	128	Take a step onto the next nutrient
117	130	inc	NE	117	**3**	**128**	Increment value in BX (*i.e.*, BX = BX+1).
118	131	if-n-equ	NE	117	3	128	BX ! = CX (yet), so execute the next instruction.
119	132	mov-head	NE	117	3	128	Restate loop by jumping to the flow head (117)

Example excerpt of the execution flow in the counting module of the evolved counting organism's genome. The execution depicted occurs on the map in Figure 1(b), starting from the star and facing north. It covers the organism taking a step forward, turning right based on the cue found there, and then taking another step forward. **Exec** is the number of instructions that have been executed, **Site** refers to the position in the genome of the instruction, **Instruction** is the Avida instruction being executed, **Facing** indicates the compass direction the organism is pointed on the grid, **Flow** is the genomic position pointed to by the flow-control head, **BX** and **CX** are the values stored in the respective registers and **Remarks** describes how the instruction operates in its current context. Values that are affected by the current instruction are in bold.

The step-counting routine is executed on both right- and left-turn paths, but the behavior it produces differs. When the organism traverses a right-turn path, the code keeps track of the forward movements of the organism, in effect measuring the distance the organism has traveled. The counting loop exit condition is set up by setting the CX register to the value of 128, by executing the get-head instruction that reads the current position of the Instruction Pointer (IP) into the CX register. Each step the organism takes in the loop is followed by an increment of the BX register. After 127 steps, the last increment of the BX register causes loop exit. Proper termination of the counting loop is essential for the organism's replication when traversing right-turn paths, since execution must reach the h-divide instruction in a code section near the end of the genome, or it will never complete its replication.

When the organism is in a left-turn environment, the counting loop monitors the number of rotations the organism executes, ensuring that it has the orientation that will keep it on the path after exiting the counting module. The organism remains in the same location and executes the loop four times, performing a 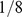 turn in each iteration. This number of iterations is determined by the sg-sense instruction (site 123, modified by the following nop-C so that the sense value is placed in the organism's CX register). When the organism is in a cell with a left-turn cue, the sg-sense instruction returns a value of 4. The value in the BX register is incremented with each loop iteration, and when the value reaches 4, execution exits the counting loop. The code section following the counting module contains another set of four 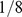 turns; the counting mechanism thus ensures that the organism will be facing forward on the path, the proper orientation to continue progress on the left-turn path. The sense value of 4 also determines how many instructions will be skipped by the jmp-head instruction. On left-turn paths, execution skips four instructions (sites 126–129), including the sg-move instruction at site 129, guaranteeing that the organism remains stationary while it corrects its orientation.

As the preceding analysis illustrates, the step-counting mechanism evolved to have interesting dynamics during execution. Instructions have different results in various environments and different instructions execute in differing conditions. To look more closely at which instructions contributed to path-following in the different environments, we ran knock-out experiments that systematically test the effect of each genome site by re-running a modified version of the organism's genome with the tested site converted to a neutral NULL instruction. We tested the organism in an ancestral environment of each type (*i.e.* right-turn and left-turn). Table 3 shows the results of these tests for the counting module, with cells containing “XXXXX” indicating that the instruction is required for the organism to successfully navigate the indicated path direction and empty cells show that the instruction does not contribute to successful task completion. Two sites (the h-copy instructions at sites 121 and 122) are not needed for either environment, six instructions contribute only in right-turn environments, two instructions are needed exclusively in left-turn environments, and six instructions are needed for both types of environments.

**Table 3:** Instructions in the step-counting module used for right- and left-turn paths.

Site	Instruction	Right paths	Left paths
117	h-search	XXXXX	XXXXX
118	sg-rotate-r	XXXXX	XXXXX
119	if-grt-0	XXXXX	
120	nop-C	XXXXX	
121	h-copy		
122	h-copy		
123	sg-sense	XXXXX	XXXXX
124	nop-C	XXXXX	XXXXX
125	jmp-head	XXXXX	XXXXX
126	sg-rotate-l	XXXXX	
127	if-equ-X	XXXXX	
128	get-head	XXXXX	
129	sg-move	XXXXX	
130	inc		XXXXX
131	if-n-equ	XXXXX	XXXXX
132	mov-head		XXXXX

Use of instructions in the step-counting module for traversing right- and left-turn paths. Cells containing “XXXXX” indicate that the instruction is necessary to complete the path following task on paths containing the indicated direction; empty cells indicate that the instruction is not used for following paths in the indicated direction.

**Figure 3:**
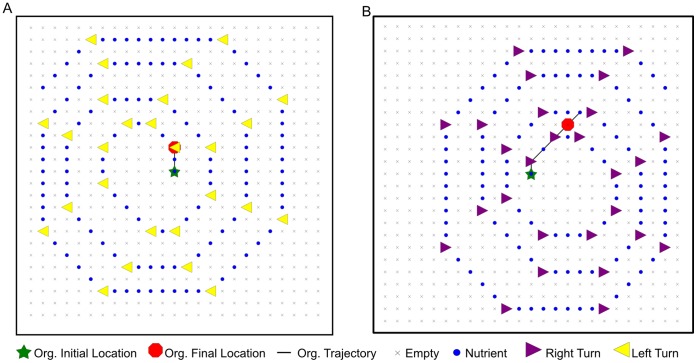
Example trajectories of the modified step-counting organism traversing ancestral paths. The increment instruction in the counting routine was replaced with a neutral instruction (nop-X). (**A**) Left-turn path. The organism stops at the first turn and fails to replicate. (**B**) Right-turn path, with extended path end. The organism misses the second turn, wanders for a couple steps, stops, but successfully completes its replication, although with a much reduced metabolic rate from the unmodified genome.

This analysis reveals an impressive degree of plasticity in the organism's execution, even in these simple, structured environments. More than half of the necessary instructions in this code segment contribute differentially to the organism's performance, depending on the current environment. Perhaps the most interesting piece of information that emerges is that the counting routine is critical for the organism to follow a left-turn path, but not for traversing right-turn paths. Since the step-counting behavior is most apparent on right-turn paths, this result seems puzzling at first. To better understand the results, we focused on one instruction in the counting sequence that is central to the counting algorithm–the increment instruction (inc). We ran execution traces of the organism in ancestral environments, replacing the inc in knock-out experiments. Trajectory traces taken from these executions are shown in Figure 3. In both left- and right-turn environments, the organism's behavior is observably poor, managing only a few steps on the path before stopping (at the first turn in the left-turn environment) or wandering off the path (in the right-turn environment). When we look at the level of the genome, we can see why inc was necessary for the path-following task in left-turn environments but not in right-turn environments. In these experiments, the path-following task is linked to replication. An organism that does not replicate will not receive a metabolic rate bonus for any part of the path-following task. In left-turn environments, the increment instruction is required to ensure loop exit from the step-counting loop so that the organism can reach the replication loop later in its genome. Without inc in the counting loop, execution is trapped in an infinite loop, and the organism cannot replicate. In right-turn environments, however, counting loop exit is triggered when the sensing instruction (sg-sense) at genome site 123 places a value of 0 in the CX register. When this event occurs, the BX register already holds a value of 0; when the comparison instruction “if-not-equal” (if-n-equ) at site 131 executes, it evaluates as false, since BX and CX both are 0. The false comparison causes execution to skip the following mov-head instruction, terminating the counting loop. Execution then continues with the instructions following the loop, eventually reaching the replication loop later in the organism's genome.

The entwining of replication and path-following also helps illuminate the behavior observed in the unfamiliar environments shown in Figure 2. In the right-turn environment of Figure 2B, the organism's backtracking behavior is triggered when it encounters a familiar pattern of cues at the beginning of the path. In the unfamiliar environment of Figure 2D, the early sections of the path do not match the familiar pattern from the ancestral environments, and the step-counting routine does not work effectively. In this situation, the organism continues to move along the path, as its execution fails to break out of the counting loop. Although the behavior appears successful at first glance, closer examination of the execution trace reveals that the organism fails to replicate in this novel situation. In the left-turn environment of Figure 2A, the organism is able to follow the trail that is modified at the end. However, as in the right-turn environment of Figure 2D, in the left-turn environment of Figure 2C the organism cannot handle changes in the pattern of cues near the beginning of the path. The organism's genome “expects” the first turn to happen after only two or three steps at the beginning of the path. When this is not the case, the organism fails to recognize the correct turn direction, and its execution enters an infinite loop as described previously.

The preceding discussion underscores that the counter was not the only feature that determined how well the organism performed. During the course of evolution, the counter became tuned to function in the context of the environments and other structures in its genome. In the next section, we look more closely at how the counter was shaped over evolutionary time.

### Evolving the Step Counter

Environmental regularities provide evolution the chance to capitalize on factors that are consistent over generational time. Since our original intent in these experiments was to probe evolving memory use [2], [3], some degree of regularity was important for our model. The population that produced the step-counting organism evolved in environments that had several regularities, some of which are inherent in the path-following problem itself. The sensory cues are the same across all generations, and the fundamental arrangement of the paths is similar (straight segments punctuated by occasional turns, with the same turn direction in any single path for the current experiments). The small set of ancestral environments meant that any individual organism had a 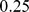 probability of being placed in the same environment as its parent, and a 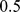 probability of being placed in the same environment type as its parent (*i.e.*, right or left). Other regularities were artifacts of development of the environments. Although unintended, these regularities could also be exploited to enhance fitness during evolution. All of the ancestral paths had short straight sections and relatively frequent turns. The ancestral right-turn paths contained 127 path steps beyond the third turn in the path. The overall path lengths varied from 130 cells to 141 cells, and the right-turn path lengths differed by only one cell (length 132 and 133). In the light of the preceding discussion of how the counting mechanism functions, it is reasonable to infer that there is a correlation between the environmental regularities and the evolved counting mechanism.

To piece together a narrative of how evolution produced the step-counting mechanism, we traced the lineage of the evolved organism back to the original ancestor of the population. We examined a number of pieces of information, including metabolic rate and fitness statistics for the line of descent, and genomes and execution traces of organisms from selected points in time. Combining all these data, we are able to reconstruct the organism's evolutionary history.

The story begins as a classic tale of gaining adaptive advantages through random mutations. In the environments of this study, the first ability an organism needs beyond replication is movement. An ancestor of our step-counting organism found that ability with a mutation in update 92, within the first 7 generations of evolution. This one mutation nearly doubled the organism's fitness. Through the next 300 or so generations, most of the progress to enhance fitness is made by gradual addition of sensing and more movement.

The step-counting mechanism was built from the inside out, by putting together two separate instructions sequences, then the loop that contained them. The instructions that would become the step-counter never resided inside the organism's copy loop. The structures in the step-counter (see Table 1) that emerged early in evolution are sites 123–125 (the sequence sg-sense/nop-C/jmp-head, which we will call the “jump sequence”) and 129–132 (sg-move/inc/if-n-equ/mov-head, which we will call the “control sequence”). These sequences are crucial to the operation of the evolved step-counter. The jump sequence serves to execute or omit instructions following the sequence based on the sensory input from the environment. The control sequence contains the increment instruction (inc) that counts the loop iterations, the comparison instruction (if-n-equ) that controls exit from the counting loop, and the mov-head instruction that marks the point of iteration.

The jump sequence begins to form in update 2700 (around generation 290) with the appearance of the jmp-head instruction. The sg-sense instruction appears in update 3322 (generation 354–364). The intervening instruction retains the original nop-C from the ancestor's genome, completing the jump sequence. This structure remains fixed through the rest of evolution. Although the jump sequence did not confer an immediate fitness advantage, it becomes crucial to the operation of the completed step-counting mechanism.

The control sequence emerges somewhat later. The several instructions that make up the sequence mutate in and out of a number of locations within the genome. In update 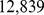 (generation 1565–1585), three simultaneous point mutations occur to produce the sequence sg-move/inc/if-n-equ. These mutations are initially deleterious, reducing the organism's fitness level to approximately 97% of its parent's fitness. At this time point, the sequence is followed by the if-grt-X instruction. That situation persists until update 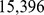, when if-grt-X mutates to mov-head, and the sequence is finished, nearly quadrupling the organism's metabolic rate. Like the jump sequence, the control sequence is preserved through the rest of evolution.

At this point in time, the two foundation sequences of the counter have appeared, but there is no loop yet to contain them. By around generation 3000 (update 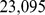), mutations have created two contiguous loops, one containing the jump sequence and the other holding the control sequence. The two loops are consolidated in update 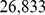 (generation 3300), establishing the essential structure of the counting loop. This change is initially neutral, with fitness remaining unchanged from parent to offspring. The structure of the loop was set, but required tuning to deliver an advantage.

The rest of the history of evolving the step-counter mechanism has two interwoven story lines, one related to slow incremental improvement of the genome to better suit the demands of the environments, and the other more focused on careful tweaks that tune the counter to both the environment and to interactions with other segments of the organism's genome. The counting loop is nearly complete at update 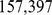 (8734 generations), when the counting loop is 15 instructions long and the same as the loop at the end of evolution except that it contains only one h-copy instruction instead of two. That final instruction is added two generations later, and the counting loop is in its final form. The organism that first has the counting code in its final form has evolved a “perfect” solution for the ancestral left-turn paths, but cannot successfully navigate the right-turn paths. The mechanism for left-turn paths emerged earlier than that for right-turn paths, reaching 84% of the maximum metabolic rate bonus by update 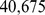 (approximately generation 5100).

Successful use of the counting loop for right-turn paths did not emerge until update 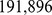 (around generation 9625). This was the first organism that was able to traverse both right- and left-turn paths. The organism's genome differs from that of its parent by one instruction inserted before the counting loop. The insertion changed the position in the genome of the get-head instruction to site 128, thus resulting in the backtracking behavior that allows the organism to complete its replication. Much of the progress between the emergence of the left-turn path strategy and the right-turn path strategy is slow incremental improvement resulting from individual mutations. This incremental improvement continues after the strategies for both path directions have emerged. The successful behavior remains the same, but fine tuning continues to deliver gradually improving metabolic rate bonuses. Deleterious mutations occasionally degrade performance for one or more generations, but successful behavior consistently reemerges. The final step-counting organism was born at update 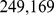 (generation 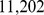), near the end of the 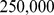 update experiment.

It is evident that the evolved step-counting mechanism is specifically adapted to the environments experienced during evolution. The evolved solution allowed a conservative strategy that produced effective fitness-enhancing behavior. Although the evolved solution is environment-specific, it could serve as the basis for a more flexible counting mechanism. The key to the way the counting algorithm works is controlling how many times the counting loop iterates. This control involves setting a register to some value that will be used to determine when to exit the loop. The existing counting method controls loop iteration with a specific sensory cue in left-turn environments and a value given by genome position in right-turn environments. A more generalized step-counter would need to control loop iterations with some arbitrary event, such as reaching a “goal” or accomplishing some behavioral task (*e.g.*, finding a food item). Imagine a scenario where the Avidian must traverse a trail to find a large food reward, then retrieve the food and retrace its steps to its starting position (its “nest”). In this situation, the organism would need to count its steps to the end of the path, then use the counter again to return home. In addition to generalizing loop iteration control, a general step-counting algorithm needs to be self-contained, minimizing interactions with other sites in the genome. The existing step-counting algorithm contains some instructions that interact with instructions elsewhere in the genome. The different operation of the counting loop in left- and right-turn environments illustrates the effects of such interactions. In left-turn environments, the counting loop counts rotations, not steps, acting as error-correction for orientation changes that occur later in the genome. In right-turn environments, the number of loop iterations is determined by the genome position of the get-head instruction. In both cases, instructions located outside of the counting module itself have a profound impact on the behavior of executing the counting loop. If the genome outside the counting loop changes, the executed behavior of the counting algorithm may in turn change dramatically. If the counting routine has fewer dependencies on other instructions, it will function more reliably and in a broader range of conditions. The evolution of a more flexible, generalized counting mechanism could occur in environments where counting, specifically step-counting, contributes strongly to increased fitness, such as the foraging scenario mentioned earlier.

### Conclusions

We have presented a detailed analysis of a complex behavior that evolved in Avida, to illustrate how we may use results such as ours to inform questions about the evolution of behavior. Even though Avida's genetic program and discrete world differ in important ways from the genes and noisy complex world of biology, results such as ours can be biologically relevant. Avida is extensible and highly configurable, allowing researchers to design experiments that probe specific hypotheses about the evolution of behavior, in the same way that other representations and methods (*e.g.*, neural networks and genetic algorithms) have been used to model hypotheses about behavior. The emergence of the step-counting mechanism in our experiments was initially a complete surprise. Because odometry was not the focus of our experiments, the experimental setup was not designed to model a problem that needed a distance measure, nor to capture data that would allow for more quantified analysis. Counting requires a fair amount of complex, sophisticated computation in Avida, and so is an excellent problem domain for investigating evolving complex behavior. In future work, we plan to focus directly on evolving odometric behavior, using Avida and other model systems. We are currently developing the design for Avida experiments that address the evolution of a flexible step-counting mechanism. Through those studies, we may shed more light on the evolutionary origin of odometry and other complex behavior.

The step-counter organism provides an excellent example of how complexity evolves, building sophisticated and specialized capabilities from the simplest components. Components that were later critical to the operation of a complex trait sometimes arose without conferring any immediate benefit or even being deleterious at first. At various periods during evolution, there were relatively sudden improvements in function compared to other periods where evolution gradually fine-tuned a trait. Of course, none of this was pre-planned; it is only in hindsight that we can trace the circuitous path that evolution followed in producing what turned out to be a useful complex trait. These results are consistent with theoretical views about how complexity evolves in nature, and show how complex behavioral traits can arise even in very simple environments without direct selection.

## References

[1] Ofria C, Bryson DM, Wilke CO (2009) Artificial life models in software. In: Adamatzky A, Komosinski M, editors, Advances in Artificial Life, Berlin: Springer-Verlag, chapter Avida: A Software Platform for Research in Computational Evolutionary Biology. 2nd edition, 3–36. 10.1162/106454604773563612 15107231

[2] Grabowski LM, Bryson DM, Dyer FC, Ofria C, Pennock RT (2010) Early evolution of memory usage in digital organisms. In: Artificial Life XII: Proceedings of the Twelfth International Conference on the Synthesis and Simulation of Living Systems. Cambridge, MA: MIT Press, 224–231.

[3] Grabowski LM, Bryson DM, Dyer FC, Pennock RT, Ofria C (2011) Clever creatures: case studies of evolved digital organisms. In: Proceedings of the Eleventh European Conference on the Synthesis and Simulation of Living Systems (ECAL 2011). Cambridge, MA: MIT Press, 276–283.

[4] Adami C, Ofria CA, Collier TC (2000) Evolution of biological complexity. Proceedings of the National Academy of Science 97: 4463–4468. 10.1073/pnas.97.9.4463 PMC18257 10781045

[5] Goldstein R (2009) Evolution of biological complexity. In: Séminaire Poincaré XII. Institut Henri Poincaré, 75–88.

[6] Lenski RE, Ofria C, Pennock RT, Adami C (2003) The evolutionary origin of complex features. Nature 423: 139–144. 12736677 10.1038/nature01568

[7] Dale K, Collett TS (2001) Using artificial evolution and selection to model insect navigation. Current Biology 11: 1305–1316. 11553323 10.1016/s0960-9822(01)00418-3

[8] Vickerstaff R, Di Paolo EA (2005) Evolving neural models of path integration. Journal of Experimental Biology 208: 3349–3366. 16109896 10.1242/jeb.01772

[9] Haferlach T, Wessnitzer J, Mangan M, Webb B (2007) Evolving a neural model of insect path integration. Adaptive Behavior 15: 273–287.

[10] Goldsby HJ, Serra N, Dyer FC, Kerr B, Ofria C (2012) The evolution of temporal polyethism. In: Artificial Life 13: Proceedings of the Thirteenth International Conference on the Simulation and Synthesis of Living Systems. Cambridge, MA: MIT Press, 178–185.

[11] Wehner R, Wehner S (1986) Path integration in desert ants: approaching a long-standing puzzle in insect navigation. Monitore Zooligico Italiano 20: 309–331.

[12] Collett TS (1992) Landmark learning and guidance in insects. Philosophical Transactions of the Royal Society of London B 337: 295–303.

[13] Wehner R (1997) The ant's celestial compass system: spectral and polarization channels. In: Lehrer M, editor, Orientation and communication in arthropods, Basel, Switzerland: Birkhauser Verlag. 145–185.

[14] Wolf H (2011) Odometry and insect navigation. Journal of Experimental Biology 214: 274–276. 10.1242/jeb.038570 21525309

[15] Wittlinger M, Wehner R, Wolf H (2006) The ant odometer: Stepping on stilts and stumps. Science 312: 1965–1967. 16809544 10.1126/science.1126912

[16] Wilke CO, Wang J, Ofria C, Adami C, Lenski RE (2001) Evolution of digital organisms at high mutation rates leads to survival of the flattest. Nature 423: 139–144. 10.1038/35085569 11460163

[17] Clune J, Misevic D, Ofria C, Lenski RE, Elena S, et al (2008) Natural selection fails to optimize mutation rates for long-term adaptation on rugged fitness landscapes. PLoS Computational Biology 4: e1000187. 18818724 10.1371/journal.pcbi.1000187 PMC2527516

[18] Misevic D, Ofria C, Lenski RE (2006) Sexual reproduction reshapes the genetic architecture of digital organisms. Proceedings of the Royal Society B 273: 457–464. 16615213 10.1098/rspb.2005.3338 PMC1560214

[19] Clune J, Goldsby HJ, Ofria C, Pennock RT (2011) Selective pressures for accurate altruism targeting: evidence from digital evolution for difficult-to-test aspects of inclusive fitness theory. Proceedings of the Royal Society B 278: 666–674. 20843843 10.1098/rspb.2010.1557 PMC3030848

[20] Ofria C, Adami C, Collier TC (2002) Design of evolvable computer languages. IEEE Transactions in Evolutionary Computation 17: 528–532.

[21] Zhang SW, Bartsch K, Srinivasan MV (1996) Maze learning by honeybees. Neurobiology of Learning and Memory 66: 267–282. 8946421 10.1006/nlme.1996.0069

